# Supplementary material for: Different Approaches for the Profiling of Cancer Pathway-Related Genes in Glioblastoma Cells
Source: Int J Mol Sci. 2022 Sep 17;23(18):10883. doi: 10.3390/ijms231810883 (PMC9504477; doi:10.3390/ijms231810883)
Supplement: Supplementary file 1 [file ijms-23-10883-s001.zip › ijms-1878548-supplementary.pdf]

Supplementary material for: *"Different approaches for the profiling of cancer pathway-related genes in glioblastoma cells"*

authors: Zuzana Majercikova, Katarina Dibdiakova, Michal Gala,
Denis Horvath, Radovan Murin, Gabriel Zoldak, Jozef Hatok

1 Methodology

1.1 Overview of supplementary material: multi-criteria decision in the analysis of gene expression profiles

In this material we present the original em multicriterial decision-making (MCDM) [1] for the possible characterization of gene expression profiles. MCDM was originally only an area of operational research, which is now increasingly influencing other domains of science. In this document we combine MCDM with constructions of disagreement measures that are significantly conditioned by data. The metric designed by us is implemented for analysis of the types of data provided by experiment. A significant feature of the data processed here is that they show commutative properties and are arranged into triplicates. The measure is complex and is significantly conditional in data. It is numerically designed to compare specific genes associated with glioblastoma profiling. Its specificity is visible in that the proposed measure contains data -related and weighted individual expression measures. Of course, there is also a significant universality of the metohodology given by the possibility of applying it to different data.

1.2 Organization of data; sets and subsets and triplicates

We analyze experimental data describing the expression properties of genes $j = 1, 2, \ldots, N_g = 67$. The structure of the data is such that there are three triplets - the nine instances of relative gene expression $I_{j,1}$, $I_{j,2}$, $\ldots$, $I_{j,9}$ in the normal tissue case as a control. The additional nine values $I_{j,10}$, $I_{j,11}, \ldots, I_{j,18}$ corresponding to three triplets are representatives of the malignancy. We denote the expression rates of the $j-$th gene by $I_{j,\ldots}$. The data organization can be described as

$$\mathbf{I}_j \equiv \begin{pmatrix} I_{j,1}, & I_{j,2}, & I_{j,3}, & I_{j,4}, & I_{j,5}, & I_{j,6}, & I_{j,7}, & I_{j,8}, & I_{j,9}, \\ I_{j,10}, & I_{j,11}, & I_{j,12}, & I_{j,13}, & I_{j,14}, & I_{j,15}, & I_{j,16}, & I_{j,17}, & I_{j,18} \end{pmatrix} . \quad \text{(S1)}$$

For better clarification and assignment of biological meaning to the genetic data, which we have chosen to be indexed as $1, \ldots, 18$, we provide the table below

$I_{\cdot,1}$ ... hRNA_1	$I_{\cdot,4}$ ... NHA_1	$I_{\cdot,7}$ ... HDFa_1	
$I_{\cdot,2}$ ... hRNA_2	$I_{\cdot,5}$ ... NHA_2	$I_{\cdot,8}$ ... HDFa_2	(S2)
$I_{\cdot,3}$ ... hRNA_3	$I_{\cdot,6}$ ... NHA_3	$I_{\cdot,9}$ ... HDFa_3	

$$\begin{array}{lll|lll|lll} I_{.,10} & \ldots & \text{T98G\_1} & I_{.,13} & \ldots & \text{A172\_1} & I_{.,16} & \ldots & \text{SW1088\_1} \\ I_{.,11} & \ldots & \text{T98G\_2} & I_{.,14} & \ldots & \text{A172\_2} & I_{.,17} & \ldots & \text{SW1088\_2} \\ I_{.,12} & \ldots & \text{T98G\_3} & I_{.,15} & \ldots & \text{A172\_3} & I_{.,18} & \ldots & \text{SW1088\_3} \end{array} .$$

Alternatively, there are sub-structures with the following sets

$$\begin{aligned} X_{1,j} &\equiv \text{Set}\,(\, I_{j,1},\, I_{j,2},\, I_{j,3}\,)\;, \\ X_{2,j} &\equiv \text{Set}\,(\, I_{j,4},\, I_{j,5},\, I_{j,6}\,)\;, \\ X_{3,j} &\equiv \text{Set}\,(\, I_{j,7},\, I_{i,8},\, I_{j,9}\,) \end{aligned} \tag{S3}$$

and

$$\begin{aligned} Y_{1,j} &\equiv \text{Set}\,(\, I_{j,10},\, I_{j,11},\, I_{j,12}\,)\;, \\ Y_{2,j} &= \text{Set}\,(\, I_{j,13},\, I_{j,14},\, I_{j,15}\,)\;, \\ Y_{3,j} &= \text{Set}\,(\, I_{j,16},\, I_{j,17},\, I_{j,18}\,)\;. \end{aligned} \tag{S4}$$

Here $X_{...}$ refers to all cases of healthy controls, whereas $Y_{...}$ refers to all malignant cases.

1.3 Inter-subset dissimilarity (inter-triplicate variations)

Let us emphasize that Set(.,.,.) is free of ordering. Thus, here we are dealing with a two-fold structure $\mathbf{I}_j \equiv (\mathbf{X}_j, \mathbf{Y}_j)$. The objective is to analyze and describe the homogeneity within the introduced tuples $\mathbf{X}_j \equiv (X_{1,j}, X_{2,j}, X_{3,j})$ and $\mathbf{Y}_j \equiv (Y_{1,j}, Y_{2,j}, Y_{3,j})$ separately, but also to explore the dissimilarities between $\mathbf{X}_j$ and $\mathbf{Y}_j$, i.e. in the terms of the composite tuple $(\mathbf{X}_j, \mathbf{Y}_j) \equiv (\mathbf{X}, \mathbf{Y})_j$. Let (U_j, V_j) be a representative of a pair of number sets in which both U_j and V_j include a triple of the real numbers. Their introduction is thus fully consistent with the data structures from Eq.(S3) and Eq.(S4). U_j, V_j are introduced as universal structures that can be used to define relations between pairs of sets in general. For each pair (U_j, V_j), we can define a transformation into three new numbers that do not depend on the order of the items inside U_j, V_j themselves. We implement this invariant generation step by selecting three basic absolute (real) differences

$$\begin{aligned} \Delta mea(U_j, V_j) &\equiv \left|\, \underset{\text{mean}}{^{\text{arithmetic}}}(U_j) - \underset{\text{mean}}{^{\text{arithmetic}}}(V_j) \,\right|\;, \\ \Delta max(U_j, V_j) &\equiv \left|\, \max(U_j) - \max(V_j) \,\right|, \\ \Delta min(U_j, V_j) &\equiv \left|\, \min(U_j) - \min(V_j) \,\right|. \end{aligned} \tag{S5}$$

The situation requires that pairwise comparisons in triples $X_{1,j}, X_{2,j}, X_{3,j}$ and $Y_{1,j}, Y_{2,j}.Y_{3,j}$ be defined. Let us now consider the combinations of the three indices $1, 2, 3$ taken two at a time. For this object, we'll utilize shortened notation $[1, 2, 3]_{\text{pair}} \equiv \{(1, 2),\, (1, 3),\, (2, 3)\}$. The pairwise comparisons within the set $\mathbf{X}_j$ provide three specific definitions

$$\begin{aligned} Dmea((\mathbf{X}, \mathbf{X})_j) &\equiv \frac{1}{3} \sum_{(k,s)\in[1,2,3]_{\text{pair}}} \Delta mea(X_{k,j}, X_{s,j})\,, \\ Dmax((\mathbf{X}, \mathbf{X})_j) &\equiv \frac{1}{3} \sum_{(k,s)\in[1,2,3]_{\text{pair}}} \Delta max(X_{k,j}, X_{s,j})\,, \\ Dmin((\mathbf{X}, \mathbf{X})_j) &\equiv \frac{1}{3} \sum_{(k,s)\in[1,2,3]_{\text{pair}}} \Delta min(X_{k,j}, X_{s,j})\,. \end{aligned} \tag{S6}$$

(The chosen normalization factor of 1/3 compensates for the number of possible terms. Because the mathematical structural features of $\mathbf{Y}_j$ are equivalent, we can write accordingly

$$\begin{aligned} Dmea((\mathbf{Y},\mathbf{Y})_j) &\equiv \frac{1}{3}\sum_{(k,s)\in[1,2,3]_{\mathrm{pair}}} \Delta mea(Y_{k,j},Y_{s,j})\,, \\ Dmax((\mathbf{Y},\mathbf{Y})_j) &\equiv \frac{1}{3}\sum_{(k,s)\in[1,2,3]_{\mathrm{pair}}} \Delta max(Y_{k,j},Y_{s,j})\,, \\ Dmin((\mathbf{Y},\mathbf{Y})_j) &\equiv \frac{1}{3}\sum_{(k,s)\in[1,2,3]_{\mathrm{pair}}} \Delta min(Y_{k,j},Y_{s,j})\,. \end{aligned} \tag{S7}$$

The following measures are used to detect differences between $\mathbf{X}_j, \mathbf{Y}_j$ without emphasis on the position of their components

$$\begin{aligned} Dmea((\mathbf{X},\mathbf{Y})_j) &= \frac{1}{9}\sum_{k=1,2,3}\sum_{s=1,2,3} \Delta mea(X_{k,j},Y_{s,j})\,, \\ Dmax((\mathbf{X},\mathbf{Y})_j) &= \frac{1}{9}\sum_{k=1,2,3}\sum_{s=1,2,3} \Delta max(X_{k,j},Y_{s,j})\,, \\ Dmin((\mathbf{X},\mathbf{Y})_j) &\equiv \frac{1}{9}\sum_{k=1,2,3}\sum_{s=1,2,3} \Delta min(X_{k,j},Y_{s,j})\,. \end{aligned} \tag{S8}$$

1.4 Mean values from dissimilarity measures

Deciding which of these measures of dissimilarity to choose is a difficult task. The different options need to be scalarized to obtain a unique output. An alternative is to take the averages of the difference measures, which can be considered a good starting point for which reductions should be made. However, this situation is complicated by the number of possible variants of the generalized mean [3] as candidates for this purpose. Therefore, the MCDM may be considered as a suitable further procedure. The latter will in fact include all candidate variant-attributes, but this is an interpretational complication. However, once the data is included, not only a comprehensive measure is offered as a result, but also with it a complete system of numeric weights, which ultimately will tell us to what extent attribute, a way of dissimilarity is averaged, is applied.

Let us be more formal and denote by $D^{(k)}$ the measure of dissimilarity that can be obtained using a suitable $k-$th mean of some primitive measures of the type $Dmea(.)$, $Dmax(.)$, $Dmin(.)$ Then introduce the appropriate scalar $g_k(.,.,.)$, which we take to be the examples of generalized mean which allows to express $D^{(k)}$ by means of algebraic functions. Assume that in the case of j-th gene there will be $(\mathbf{X},\mathbf{X})_j$, $(\mathbf{Y},\mathbf{Y})_j$, $(\mathbf{X},\mathbf{Y})_j$ there are pairs characterized by the dissimilarity measures

$$\begin{aligned} D^{(k)}((\mathbf{X},\mathbf{X})_j) &= g_k\left(Dmea((\mathbf{X},\mathbf{X})_j), Dmax((\mathbf{X},\mathbf{X})_j), Dmin((\mathbf{X},\mathbf{X})_j)\right)\,, \\ D^{(k)}((\mathbf{Y},\mathbf{Y})_j) &= g_k\left(Dmea((\mathbf{Y},\mathbf{Y})_j), Dmax((\mathbf{Y},\mathbf{Y})_j), Dmin((\mathbf{Y},\mathbf{Y})_j)\right)\,, \\ D^{(k)}((\mathbf{X},\mathbf{Y})_j) &= g_k\left(Dmea((\mathbf{X},\mathbf{Y})_j),\ Dmax((\mathbf{X},\mathbf{Y})_j),\ Dmin((\mathbf{X},\mathbf{Y})_j)\right)\,. \end{aligned} \tag{S9}$$

From a practical point of view, we suggested list containing $g_1(.)$, $g_2(.)$, $\ldots$ $g_{N_c}(.)$ functions, which represent the attributes of MCDM. The specific list of generalized means (including units indexed

up to $N_c = 11$) employed for our purposes includes

$$\begin{aligned}
g_1(a,b,c) &= \frac{1}{3}(a+b+c)\,, \\
g_2(a,b,c) &= (\,abc\,)^{\frac{1}{3}}\,, \\
g_3(a,b,c) &= \frac{1}{3}\left(\sqrt{ab}+\sqrt{ac}+\sqrt{bc}\right)\,, \\
g_4(a,b,c) &= \left[\frac{(a+b)}{2}\,\frac{(a+c)}{2}\,\frac{(b+c)}{2}\right]^{\frac{1}{3}}\,, \\
g_5(a,b,c) &= \left[\frac{1}{3}\left(a^{-1}+b^{-1}+c^{-1}\right)\right]^{-1}\,, \\
g_6(a,b,c) &= \left[\frac{1}{3}\left(a^{-2}+b^{-2}+c^{-2}\right)\right]^{-\frac{1}{2}}\,, \\
g_7(a,b,c) &= \left[\frac{1}{3}\left(a^{-3}+b^{-3}+c^{-3}\right)\right]^{-\frac{1}{3}}\,, \\
g_8(a,b,c) &= \sqrt{\frac{1}{3}(a^2+b^2+c^2)}\,, \\
g_9(a,b,c) &= \left[\frac{1}{3}(a^3+b^3+c^3)\right]^{\frac{1}{3}}\,, \\
g_{10}(a,b,c) &= \frac{a^2+b^2+c^2}{a+b+c}\,, \\
g_{11}(a,b,c) &= \frac{a^3+b^3+c^3}{a^2+b^2+c^2}\,.
\end{aligned} \tag{S10}$$

Here a, b, c represent an arbitrary nonzero positive arguments.

1.5 Weighting and relative measures of inter-subset differences

In cases where we want to consider the differences of $\mathbf{X}$ and $\mathbf{Y}$ with respect to the similarities in the $\mathbf{X}$ frame or also in the $\mathbf{Y}$ frame, we obtain, for example, the definition of a dimensionless measure

$$R_j^{(k)} = \frac{D^{(k)}((\mathbf{X},\mathbf{Y})_j)}{\sqrt{D^{(k)}((\mathbf{X},\mathbf{X})_j)\,D^{(k)}((\mathbf{Y},\mathbf{Y})_j)}} \tag{S11}$$

defined in connection to the $j-$th gene projected on the $k-$th attribute - mean, is fundamental to our efforts. If we apply all the attributes to all the genes (N_g), we get summary, basic statistics

$$\sigma_R^{(k)} = \sqrt{\frac{1}{N_g-1}\sum_{j=1}^{N_g}\left(R_j^{(k)}-\overline{R}^{(k)}\right)^2}\,, \qquad \overline{R}^{(k)} = \frac{1}{N_g}\sum_{j=1}^{N_g} R_j^{(k)}\,. \tag{S12}$$

The standard focus of the MCDM is the calculation of weights of attributes. They can be determined using

$$w_R^{(k)} = \frac{\sigma_R^{(k)}}{\sum_{k'=1}^{N_c}\sigma_R^{(k')}}\,. \tag{S13}$$

Therefore, the role of the average g_k with higher variance is preferred. The following weighted average is given by

$$\tilde{R}_j = \sum_{k=1}^{N_c} w_R^{(k)} R_j^{(k)} \,. \tag{S14}$$

We will subsequently extend this measure. The extension is based on the approximation of the weights retained.

1.6 Over- and under expression properly characterized

To make the description more detailed, we abandon the formulation in terms of the absolute values. We are instead interested in a description using the pair of functions

$$|\,x\,|^{+} \;=\; \begin{cases} x & \text{for} \quad x > 0 \\ 0 & \text{for} \quad x \leq 0 \end{cases} \,, \qquad \big(\text{emphasis on over-expression}\big) \tag{S15}$$

$$|\,x\,|^{-} \;=\; \begin{cases} -x & \text{for} \quad x < 0 \\ 0 & \text{for} \quad x \geq 0 \end{cases} \qquad \big(\text{emphasis on under-expression}\big)\,. \tag{S16}$$

The plus/minus symbols are indicative only, they are not operations. They only serve to extend the definition of absolute value by focusing it on two different areas, thus obtaining two variants of functions. It is convenient to redefine and modify the original Δmea, Δmax, Δmin and thus obtain their more structured alternatives

$$\begin{aligned} \Delta^{\pm} mea(U_j, V_j) \;&\equiv\; \Big|\, \underset{\text{mean}}{\text{arithmetic}}(U_j) - \underset{\text{mean}}{\text{arithmetic}}(V_j) \Big|^{\pm} \,, \\ \Delta^{\pm} max(U_j, V_j) \;&\equiv\; \big|\, \max(U_j) - \max(V_j) \,\big|^{\pm} \,, \\ \Delta^{\pm} min(U_j, V_j) \;&\equiv\; \big|\, \min(U_j) - \min(V_j) \,\big|^{\pm} \,. \end{aligned} \tag{S17}$$

The same logic can be used when defining intermediate steps within auxiliary expressions $D^{\pm}mea$, $D^{\pm}max$, $D^{\pm}min$. The original, which is given by Eq.(S6), then changes to

$$Dmea^{\pm}((\mathbf{X},\mathbf{X})_j) \;\equiv\; \frac{1}{3} \sum_{(k,s)\in[1,2,3]_{(2)}} \Delta^{\pm} mea(X_{k,j}, X_{s,j})\,. \tag{S18}$$

Analogously we obtained the diversity measures $D^{\pm,(k)}((\mathbf{X},\mathbf{Y})_j)$ which finally yield definition

$$R_j^{\pm,(k)} = \frac{D^{\pm,(k)}((\mathbf{X},\mathbf{Y})_j)}{\sqrt{D^{(k)}((\mathbf{X},\mathbf{X})_j)\, D^{(k)}((\mathbf{Y},\mathbf{Y})_j)}} \,. \tag{S19}$$

(The denominator of the expression shown is not incorrect, it is left as originally defined because it does not focus on the relationship between control and between diseased tissues.) We turn to the canonical aspect of MCDM to use the approximation, where weights are obtained for $R_j^{\pm,(k)}$ to calculate

$$\tilde{R}_j^{\pm} = \sum_{k=1}^{N_c} w_R^{(k)} R_j^{\pm,(k)} \,. \tag{S20}$$

Note that for the figures in the main text, the reduced notation R^{+}, R^{-} is used to label the corresponding means of $R^{\pm,(k)}$ values with the respective weights.

2 Results for data-driven weights

The system of weights associated with Eq.(S20) obtained for the experimental data is

$$\begin{aligned} w_R^{(1)} &= 0.0712\,,\ w_R^{(2)} = 0.0854\,,\ w_R^{(3)} = 0.0789\,,\ w_R^{(4)} = 0.0723\,, \\ w_R^{(5)} &= 0.1210\,,\ w_R^{(6)} = 0.1478\,,\ \underline{w_R^{(7)} = 0.1611}\,,\ w_R^{(8)} = 0.0676\,, \\ w_R^{(9)} &= 0.0662\,,\ w_R^{(10)} = 0.0644\,,\ \underline{w_R^{(11)} = 0.0636}\,. \end{aligned} \tag{S21}$$

From these results it is evident that the exceptional weights are as follows

1. Maximum $w_R^{(7)}$, which corresponds to $[(1/3)(a^{-3} + b^{-3} + c^{-3})]^{-1/3}$, which can be called *third order superharmonic mean.*

2. Minimum $w_R^{(11)}$ belongs to $\frac{a^3+b^3+c^3}{a^2+b^2+c^2}$, which is Lehmer mean [2] of power 3. Note also that several generalized averages ($k = 8, 9, 10$) are close in the magnitude to 0.06.

References

[1] B. Paradowski, A. Shekhovtsov, A. Baczkiewicz, B. Kizielewicz, W. Salabun, *Similarity Analysis of Methods for Objective Determination of Weights in Multi-Criteria Decision Support Systems*. Symmetry 2021, **13** 1874. https://doi.org/10.3390/sym13101874

[2] E.W. Weisstein, *Lehmer Mean. From MathWorld, A Wolfram Web Resource.* https://mathworld.wolfram.com/LehmerMean.html

[3] P. S. Bullen: *Handbook of Means and Their Inequalities*. Dordrecht, Netherlands: Kluwer, 2003, pp. 175-177
